# Emergence of antibiotic-resistant pneumococcal serotypes causing invasive pneumococcal disease in children, Spain

**DOI:** 10.1128/aac.01530-25

**Published:** 2025-12-31

**Authors:** Joaquín Llorente, Julio Sempere, Mirella Llamosí, Covadonga Pérez-García, Aída Úbeda, Erick Joan Vidal-Alcántara, Juan Carlos Sanz, Mirian Domenech, Jose Yuste

**Affiliations:** 1 Spanish Pneumococcal Reference Laboratory, National Center for Microbiology, Instituto de Salud Carlos III 38176, Madrid, Spain; 2 Doctoral Program in Biomedical Sciences and Public Health, Spanish University of Distance Education (UNED) 572571, Madrid, Spain; 3 Clinical Unit of Infectious Diseases, Microbiology and Parasitology, Virgen del Rocio University Hospital. Institute of Biomedicine of Seville (IBIS) 16885, Seville, Spain; 4 CIBER de Enfermedades Respiratorias (CIBERES), Instituto de Salud Carlos III 38176, Madrid, Spain; 5 Regional Public Health Laboratory, Comunidad de Madrid 37143, Madrid, Spain; 6 CIBER de Epidemiología y Salud Pública (CIBERESP), Instituto de Salud Carlos III 91837 , Madrid, Spain; University Children’s Hospital Münster, Münster, Germany

**Keywords:** invasive pneumococcal diseases, pneumococcal conjugated vaccine, resistant antibiotics, serotype replacement

## Abstract

Pneumococcal conjugate vaccines (PCVs) have significantly reduced disease burden caused by *Streptococcus pneumoniae*, a leading cause of childhood morbidity and mortality globally. The rise of non-vaccine serotypes is a frequent phenomenon after the use of these PCVs. This study is a national surveillance that includes all pneumococcal isolates causing invasive pneumococcal disease (IPD) (4,455 isolates) in the pediatric population to analyze the changes of strains with reduced susceptibility (IPD-RS) to different antibiotics (1,458 to penicillin/1,304 to erythromycin) and the impact of PCVs and COVID-19 pandemic on antibiotic resistance. Six periods are differentiated according to this decline: pre-PCV13, early PCV13, middle PCV13, late PCV13, COVID-19, and reopening. Between 2009 and 2023, overall IPD cases in Spain decreased by over 60% in children aged 1–4 years and by approximately 50% in infants under 1 year of age. Nevertheless, an increase in IPD-RS caused by non-PCV13 serotypes was observed, with serotype 24F being the most prevalent, which is not included in the currently licensed PCVs. The introduction of PCV13 showed a substantial impact on reducing IPD in children. The COVID-19 pandemic led to a temporary decline in the burden of disease caused by resistant strains in 2020 due to non-pharmacological measures followed by a subsequent recovery.

## INTRODUCTION

*Streptococcus pneumoniae* (pneumococcus) is responsible for >300 000 deaths globally each year in children <5 years old and has a broad clinical spectrum, from mucosal colonization to serious diseases such as pneumonia and invasive pneumococcal disease (IPD), including meningitis, bacteremia, sepsis, and others (1). IPD and pneumococcal pneumonia are major causes of morbidity and mortality in young children worldwide (1). Due to the introduction of pneumococcal conjugate vaccines (PCVs) in children, the incidence of IPD and pneumococcal pneumonia has decreased in recent years in many countries (2–5). In Spain, the incidence rate of IPD in children under 2 years of age from 2022 to 2023 was 27.1 cases per 100,000 inhabitants (2). The use of PCV13 has demonstrated a marked reduction in the incidence of IPD caused by vaccine serotypes not only in children but also in adults due to herd effects by the pediatric vaccination (6). Moreover, the use of PCVs in Spain reduced the burden of disease caused by vaccine-preventable multidrug-resistant serotypes in adults (7). This change in the epidemiology of serotypes causing IPD makes it essential to monitor the trends in the coming years for the introduction of future broader vaccines.

In Spain, PCV7 was approved in 2001 and scarcely used in the private market. Later, in 2010, PCV13 was introduced in the private market with vaccination rates around 70% until 2016 when it was funded by the national health system and included at the pediatric vaccination calendar using a 2+1 schedule (6). In 2023/2024, two new PCVs have been approved, PCV15 and PCV20. In Spain, these new PCVs have been included in the national vaccination calendar with variations depending on the Spanish region, as some regions have chosen PCV15 at 2+1 schedule, whereas other regions have implemented PCV20 at 3+1 schedule. One of the most important aspects of PCVs is that they have also contributed to controlling the spread of antibiotic resistance worldwide (8–10). PCVs have reduced the incidence of vaccine serotypes with reduced susceptibility to antibiotics (8–10). However, a major limitation of PCVs is that after the introduction of these PCVs, the rise of non-vaccine serotypes is a great concern in public health, known as serotype replacement. This is especially worrisome in the case of non-covered serotypes displaying antibiotic resistance (11, 12). The COVID-19 pandemic in 2020, along with the introduction of non-pharmaceutical interventions such as face masks, social distancing, and the nationwide lockdown implemented in Spain from March to June, clearly affected the transmission of pneumococcal strains (13). During the first 2 years of the COVID-19 pandemic, a general reduction in IPD cases was reported in several countries worldwide, including Spain, followed by an increasing trend toward the end of 2021 (5, 13).

In this study, we have analyzed the impact of the PCV13 vaccine and the COVID-19 pandemic on the circulation of serotypes with reduced susceptibility to antibiotics in children in Spain from 2009 to 2023. In addition, we have characterized the distribution of the most prevalent serotypes associated with antibiotic resistance in children and the potential coverage of current PCVs against resistant strains.

## RESULTS

### Changes in the incidence of pediatric IPD

Based on the corrected IPD incidence in the pediatric population, we observed a reduction of the burden of the pediatric IPD among children under 1 year of age (2023 vs 2009 IRR, 0.76; 95% CI, 0.57–1.01), children 1 to <2 years (2023 vs 2009 IRR, 0.48; 95% CI, 0.35–0.67), and children between 2 and 4 years (2023 vs 2009 IRR, 0.45; 95% CI, 0.34–0.59) (Table 1). This downward trend becomes notably evident starting in 2013, marking the beginning of the middle PCV13 period, during which the incidence of IPD cases declined by approximately 50% across all age groups between 0 and 5 years.

**TABLE 1:** Pediatric corrected IPD incidence stratified by age group and vaccination coverages

Periods		Pre- PCV13	Early PCV13			Middle PCV13				Late PCV13			COVID-19		Reopening		IRR 2023 vs 2009 (95% CI)
Corrected IPD incidence (100,000)	Age (years)	2009	2010	2011	2012	2013	2014	2015	2016	2017	2018	2019	2020	2021	2022	2023	
Total	<1	35.89	32.00	22.00	20.82	19.98	20.59	20.87	16.79	24.40	27.46	31.21	16.76	12.40	22.61	27.28	0.76 (0.57–1.01)
	1 to <2	36.00	26.58	20.75	14.76	15.78	13.84	23.07	16.15	15.77	21.55	15.17	8.32	15.68	24.95	17.29	0.48 (0.35–0.67)
	2–4	17.54	14.55	11.62	9.72	7.77	6.49	6.96	7.05	7.62	7.66	7.58	3.56	4.97	9.90	7.89	0.45 (0.34–0.59)
	≥5	3.81	2.43	2.06	1.81	1.63	1.58	1.33	1.32	1.01	1.31	1.34	0.59	0.36	1.74	1.42	0.37 (0.27–0.51)
Reduced susceptibility to penicillin	<1	12.39	10.13	8.15	5.63	6.37	6.76	5.52	5.91	9.82	7.28	8.07	4.74	2.33	4.68	9.09	0.73 (0.45–1.20)
	1 to <2	13.44	16.19	9.62	7.25	5.62	6.92	10.35	7.63	6.84	5.77	4.74	3.99	8.02	8.81	7.57	0.56 (0.34–0.93)
	2–4	4.41	4.23	3.21	3.38	2.92	2.60	3.39	3.16	3.05	3.68	3.40	1.39	1.97	3.37	3.94	0.89 (0.58–1.38)
	≥5	0.26	0.39	0.36	0.27	0.29	0.26	0.31	0.29	0.21	0.26	0.34	0.18	0.13	0.21	0.45	1.75 (0.78–3.92)
Resistant to erythromycin	<1	10.07	10.40	6.79	5.35	5.79	5.53	5.22	5.60	9.19	7.61	8.42	5.83	1.16	7.41	12.12	1.20 (0.75–1.92)
	1 to <2	13.44	15.46	8.61	7.51	5.62	5.81	8.87	6.46	6.54	6.98	6.95	4.66	5.58	10.64	8.29	0.62 (0.38–1.01)
	2–4	3.20	4.48	2.89	2.47	1.67	1.39	1.78	1.76	2.38	3.10	2.72	1.48	2.38	3.05	3.72	1.16 (0.73–1.86)
	≥5	0.31	0.39	0.41	0.27	0.19	0.13	0.10	0.16	0.23	0.15	0.21	0.13	0.10	0.18	0.45	1.43 (0.67–3.05)
Vaccination coverage, two doses (%)		Data unknown								94.95	97.69	97.46	97.74	97.24	98.02	98.62	
accination coverage, booster (**%**)										88.46	94.79	94.42	93.71	93.24	94.88	96.54	

In total, 1,458 isolates (32.73% of all IPD cases) with reduced susceptibility to penicillin (IPD-RSP) were identified from 2009 to 2023. During the pre-PCV13 period, the highest incidence of IPD-RSP occurred in the pediatric population 1 to <2 years of age (13.44 per 100,000). However, by 2023, the highest incidence of IPD-RSP was observed in children under 1 year old (9.09 per 100,000) (Table 1). Regarding isolates with erythromycin-resistant or resistant to macrolides (IPD-ER), a total of 1,304 isolates were evaluated. In the pre-PCV13 period, again the 1 to <2 years age group showed the highest incidence. However, at the beginning of the PCV13 middle period, a substantial decline in IPD-ER incidence was observed in this age group, as well as among infants under 1 year, a trend that persisted until the COVID-19 period (Table 1). In 2023, the incidence of IPD-ER cases returned to pre-PCV13 levels in both infants under 1 year (2023 vs 2009 IRR, 1.20; 95% CI, 0.75–1.92) and children aged 2–4 years (2023 vs 2009 IRR, 1.16; 95% CI, 0.73–1.86), whereas in the 1 to <2 years group, we observed an almost significant reduction in incidence (2023 vs 2009 IRR, 0.62; 95% CI, 0.38–1.01). Throughout the entire 15-year study period, the ≥5 years age group consistently recorded the lowest number of incidence for both IPD-RSP and IPD-ER (Table 1).

**Fig 1:**
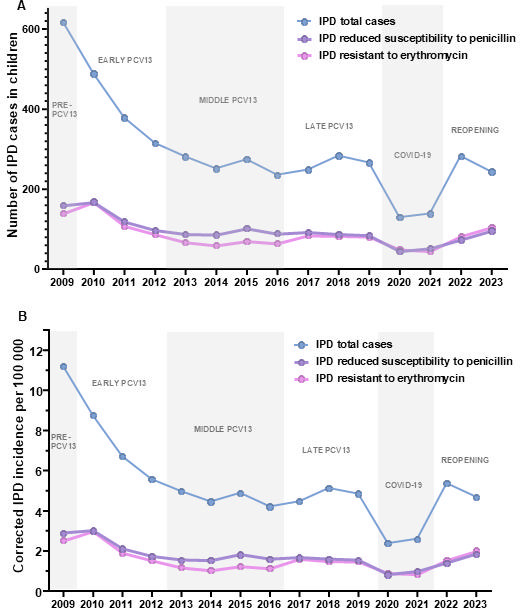
Total number of IPD cases of children under 15 years of age in Spain. Data show the total number of IPD cases included in our study (**A**), the total IPD (blue line), the IPD-RSP (purple line), and the IPD-ER (pink line). There are different periods of the study in relation to the implementation of the PCV13 in the national health system of Spain. The incidence of cases per 100,000 inhabitants in Spain (**B**) is also shown by dividing them into total IPD (blue line), IPD-RSP (purple line), and IPD-ER (pink line). There are legends in the graph to identify the different periods of the study in relation to the implementation of the PCV13 vaccine in the national health system of Spain.

The annual distribution of these cases depends on the PCV13 vaccination period (Fig. 1), a significant decline in the total number of IPD cases was observed, from 618 cases in 2009 (pre-PCV13 period) to 267 cases in 2019 (late PCV13 period), with the lowest number of cases at the end of the middle period (236 cases in 2016). Notably, the higher decline occurred during the early period, with the total number of pediatric IPD cases reduced by 50% in 2013 (Fig. 1A). In the early PCV13 period, the decline was very marked, dropping to almost 50% of IPD-RSP cases in 3 years (169 in 2010 compared to 89 in 2013), whereas in the middle-PCV13 and late periods, the number of cases remained stable. The arrival of the COVID-19 pandemic led to an overall decrease of IPD-RSP and IPD-ER cases and incidence (Fig. 1). In the following years of the pandemic, the number of cases increased while barrier measures were eliminated at the population level. Since the introduction of PCV13, the incidence of all IPD in children under 15 years old has markedly declined from 11.2 cases per 100,000 to 4.7 cases per 100,000 in 2023 (2023 vs 2009 IRR, 0.41; 95% CI, 0.36–0.48) (Fig. 1B). However, we observed a moderate reduction for IPD-RSP (2023 vs 2009 IRR, 0.63; 95% CI, 0.49–0.81) and IPD-ER (2023 vs 2009 IRR, 0.78; 95% CI, 0.61–1.01) (Fig. 1B).

### Changes in the incidence of pediatric IPD according to antibiotic susceptibility

**Fig 2:**
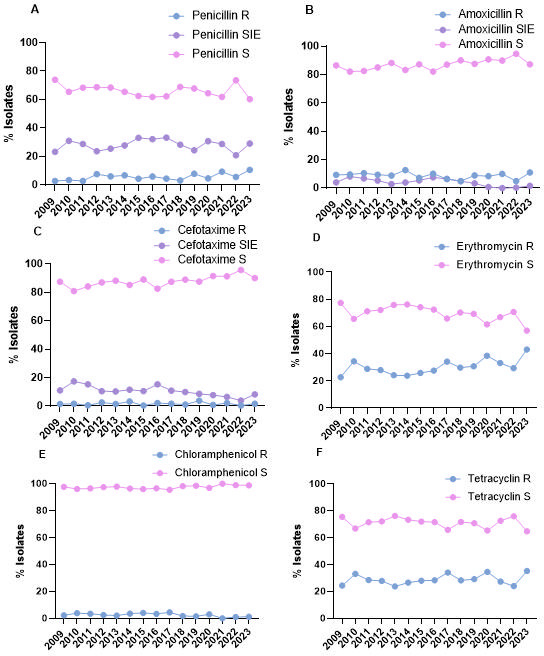
Antibiotic susceptibility of IPD cases in children under 15 years of age in Spain. Data show the percentage of IPD with resistance to penicillin (**A**), amoxicillin (**B**), cefotaxime (**C**), erythromycin (**D**), chloramphenicol (**E**), and tetracyclines (**F**). According to EUCAST 2023 criteria, they are classified as resistant (blue line), susceptible at increased exposure (purple line), and susceptible (pink line). The study period spans 2009 and 2023. R, resistant; S, susceptible; SIE, susceptible with increased exposure.

**Fig 3:**
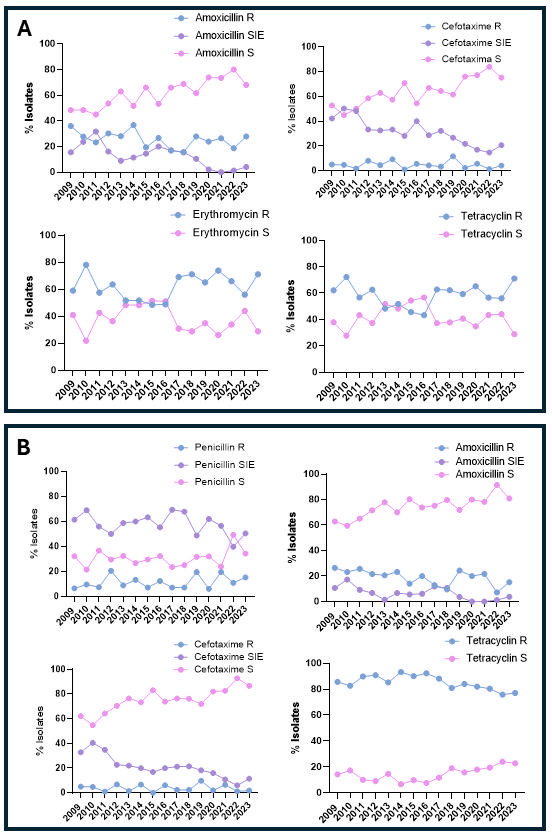
Multidrug resistance in children under 15 years of age in Spain. (**A**) IPD-RSP strains with susceptibility patterns to other antibiotics and (**B**) IPD-ER strains with susceptibility patterns to other antibiotics. Data show the percentage of IPD cases with resistance to amoxicillin, cefotaxime, erythromycin, and tetracyclines. According to EUCAST 2023 criteria, they are classified as resistant (blue line), susceptible at increased exposure (purple line), and susceptible (pink line). The study period spans 2009 and 2023. R, resistant; S, susceptible; SIE, susceptible with increased exposure.

Antibiotic susceptibility of all pediatric IPD isolates was analyzed over the 15-year study period (Fig. 2). No significant changes were observed in the trends of susceptibility to the different antibiotics analyzed over the study period. The majority of isolates were susceptible to amoxicillin, cefotaxime, and chloramphenicol (Fig. 2B, C and E). In the case of macrolides and tetracyclines, a higher percentage of resistant isolates were observed throughout the study period, with curves similar to those recorded for isolates with reduced susceptibility to penicillin (Fig. 2A, D and F). When we analyzed the changes in the antibiotic susceptibility of cases by IPD-RSP, excluding chloramphenicol due to its low clinical use and levofloxacin because it is not recommended for use in children, the antibiotics with the highest percentage of susceptible strains are β-lactams, specifically amoxicillin and cefotaxime (Fig. 3A). When analyzing the changes of susceptibility to erythromycin and tetracycline within IPD-RSP isolates, a decrease in macrolide- and tetracycline-resistant isolates was observed during the early and middle PCV13 periods. However, in 2023, resistance levels surpassed those recorded in the pre-PCV13 period (2023 vs 2009, *P* = 0.0677 for erythromycin and *P* = 0.1798 for tetracycline, chi-square test) (Fig. 3A). The percentage of isolates resistant to erythromycin and penicillin was the only one to increase by the end of the study period (Fig. 3B). In IPD-ER isolates, an increase in strains susceptible to amoxicillin and cefotaxime has once again been observed since the introduction of PCV13 (2023 vs 2009, *P* = 0.0069 for amoxicillin and *P*< 0.0001 for cefotaxime, chi-square test) (Fig. 3B).

The MIC_90_ values have remained generally stable for all antibiotics analyzed. Although the MIC_90_ for cefotaxime and amoxicillin shifted to within the susceptible range during the COVID-19 and reopening periods (Table S1). When analyzing the MIC_90_ values in IPD-RSP and IPD-ER strains, a stability of values is observed over the 15-year study period (Table S1).

### Characterization of the pneumococcal serotype distribution

**Fig 4:**
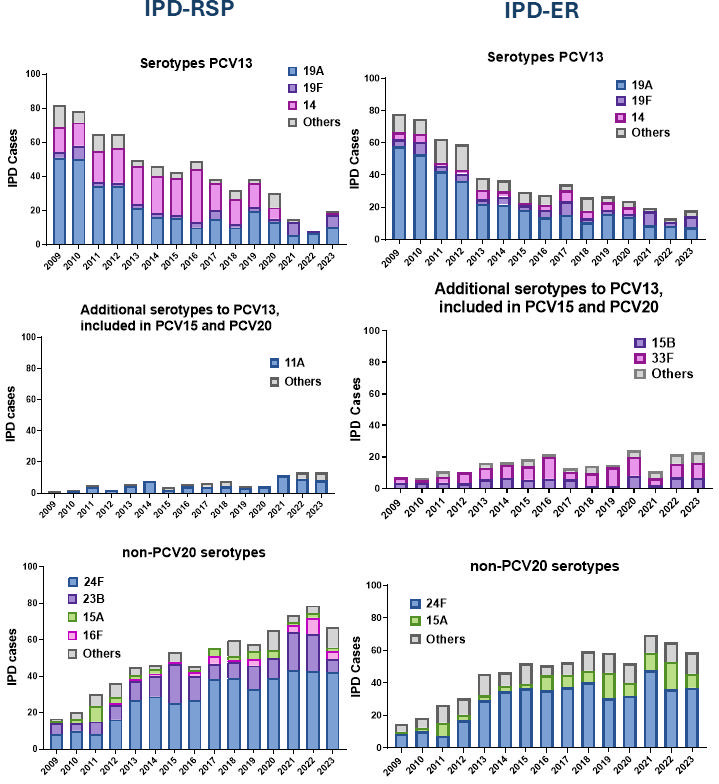
Distribution of *S. pneumoniae* serotypes causing IPD according to their inclusion in PCV13, PCV15, and/or PCV20. Data show that the cases of IPD-RSP (panels on the left) are represented in three graphs: if they are due to serotypes included in the PCV13 (top), if they are included in the PCV15 or PCV20 (middle), and those not included in these vaccines (bottom). In all groups, the most prevalent serotypes are highlighted. The cases of IPD-ER (panels on the right) are represented in three graphs: if they are due to serotypes included in the PCV13 vaccine (top), if they are included in the PCV15 or PCV20 (middle), and those not included in these vaccines (bottom). In all groups, the most prevalent serotypes are highlighted.

The slight decrease observed in the number of cases of IPD-RSP was due to the decrease in cases caused by serotypes included in PCV13 (Fig. 4). IPD-RSP due to PCV13 serotypes declined from 81.99% cases in 2009 to 19.59% in 2023. From 2009 to 2020, the main PCV13 serotypes identified in IPD-RSP isolates were serotype 19A, followed by serotypes 14 and 19F. However, in the reopening, serotypes 19A and 19F were the most prevalent, and serotype 14 disappeared. Additional serotypes included in PCV15 and PCV20, in 2023, represented 13.4% of total cases of IPD-RSP, with serotype 11A being the most prevalent among IPD-RSP isolates. However, we observed an increase in non-PCV serotypes associated with resistance in recent years, reaching 67.01% in 2023. Specifically, a progressive increase of serotype 24F was observed, followed by serotypes 23B, 15A, and 16F. Notably, 16F has gained importance in terms of case numbers during the last 3 years of the study (Fig. 4).

IPD-ER isolates exhibited a similar trend, with a decline in cases that was mainly attributable to the reduction of PCV13 serotypes (Fig. 4). The main serotypes responsible for IPD in ER isolates were 19A, followed by 19F and 14. Notably, serotype 14 was no longer detected from 2021 to 2023. Regarding the prevalence of serotypes included in PCV15 and PCV20, serotypes 33F and 15B were the most common. Finally, non-PCV serotypes associated with IPD-ER cases were primarily serotype 24F, followed by serotype 15A (Fig. 4).

The four most prevalent serotypes associated with reduced antibiotic susceptibility causing IPD were serotypes 11A, 19F, 19A, and 24F. Among these serotypes, serotype 24F exhibited the lowest MIC_90_ to β-lactams throughout the study period. For serotypes 19F and 11A, an increase in MIC_90_ was observed for penicillin/cefotaxime/amoxicillin in 19F and for penicillin in 11A during the COVID-19 pandemic and/or reopening periods. The MIC_90_ for chloramphenicol and levofloxacin remained stable throughout the 15-year study period, which is expected since these antibiotics are not used in the pediatric population (Table S2).

### Impact of pneumococcal vaccination on the pediatric population

We evaluated the potential coverage of different PCVs against IPD cases in the pediatric population during the study period (2009–2023).

**Fig 5:**
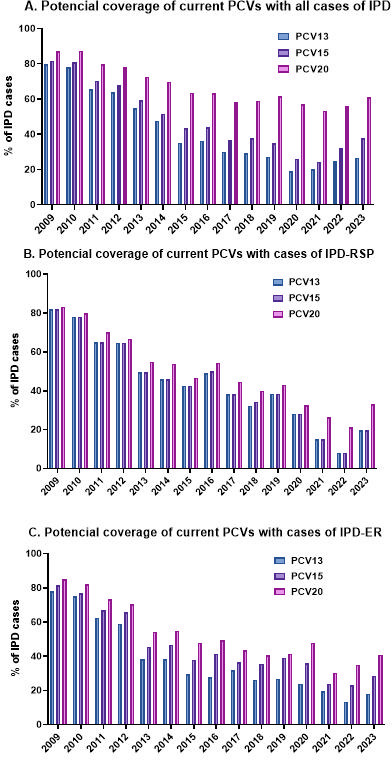
Fraction of IPD that may be prevented by different PCVs. Data show that the potential coverage of the different PCVs in children under 15 years of age in Spain, the total IPD (**A**), the IPD-RSP (**B**), and the IPD-ER (**C**). There are three bars that differentiate the IPD included in PCV13 (blue line), those included in PCV15 (purple line), and PCV20 (pink line).

In the early years, PCV13 was almost as protective as the broader-spectrum PCV15 and PCV20 would have been. However, over time, the phenomenon of serotype replacement led to a gradual improvement in coverage by the newer vaccines, particularly PCV20, due to the inclusion of additional serotypes (Fig. 5). If we focus on the last year of study, we could observe that PCV15 would potentially avoid between 26.64% and 37.70% more IPD cases in comparison to PCV13. However, the use of PCV20 would prevent between 26.64% and 61.07% more IPD cases than PCV13 in both total IPD and IPD-ER (Fig. 5). On the other hand, PCV15 would provide the same level of protection as PCV13 against IPD-RSP isolates, but more protection against IPD-ER cases as 33F only associated with macrolide resistance. In this case, PCV20 would have improved protection compared to PCV13, both for IPD-RSP and IPD-ER cases.

## DISCUSSION

Microbiological epidemiological surveillance of IPD is important to evaluate the dynamic distribution of serotypes (vaccine and non-vaccine) that are useful to evaluate the effectiveness of pediatric immunization calendars in reducing the burden of disease (14, 15). The detection of emerging serotypes not targeted by current vaccines is also another crucial aspect of surveillance programs that may guide the selection of serotypes for future vaccine formulations. Prevention strategies based on the immunization with PCVs in children and adults seem to be the best measure to control the development of IPD and reduce the impact of antimicrobial resistance (6, 7, 9, 16–18). Our study showed dynamic trends in IPD-RSP and IPD-ER in children in Spain from 2009 to 2023 covering the pre-pandemic landscape and the impact of COVID-19. The results confirm that the use of PCV13 in the pediatric population reduced IPD cases by 60.6% in Spain, and with the COVID-19 pandemic, cases decreased even further compared to the middle-PCV13 period. The decrease in the total number of IPD-RSP is associated mainly with cases caused by PCV13 serotypes, as in other described studies (2, 19). Serotypes 19F and 19A were identified as the most prevalent PCV13 serotypes associated with antibiotic resistance. These serotypes have historically played a significant role in the epidemiology of IPD in both pediatric and adult populations, contributing substantially to disease burden. However, following the widespread introduction of PCVs, the involvement of these serotypes in IPD cases has shown a marked decline. This reduction likely reflects the impact of vaccine-driven shifts in serotype circulation, as it has been found in other countries, highlighting the effectiveness of PCVs in controlling disease caused by previously dominant, resistant serotypes (20). From 2010 to 2012, which we consider the early period of vaccination with PCV13 in Spain, the decrease in IPD-RSP caused by serotypes included in PCV13 is very marked; a similar phenomenon is described in a study in the adult population in Spain (7). From 2013 to 2019 (middle and late PCV13 period), the percentage of IPD-RSP by serotypes included in PCV13 remains stable. The trends in antimicrobial resistance to erythromycin and tetracycline observed in our study are consistent with previous reports analyzing both non-IPD and IPD *S. pneumoniae* isolates from a comparable period (2007–2023) (21). This concordance may be explained by differences in serotype distribution, particularly the prevalence of serotypes such as 14, 19F, 19A, and 24F, which frequently harbor concurrent resistance to macrolides and tetracyclines (22).

Finally, in the COVID-19 pandemic period, there was a marked decline. PCV13 was effective in preventing IPD-RSP in children, although we observed serotype replacement by non-PCV13 serotypes. Several countries have also reported replacement by non-vaccine types including those associated with multidrug-resistant isolates (5, 23–25). Some of the most prevalent non-PCV13 serotypes that are included in newer PCVs were 33F (PCV15 serotype) for macrolide resistance and 11A (PCV20 serotype) for resistance to penicillin and other beta-lactam. In Spain, as well as in other European countries, the multidrug-resistant phenotype identified in serotype 11A pneumococci appears to be linked to the emergence and spread of a vaccine-escape lineage derived from genetic recombination within the globally disseminated PMEN3 clone (CC156-GPSC6). Its invasive potential has been previously documented in other countries, suggesting that recombination events in this successful clone not only facilitated serotype replacement but also contributed to the persistence of multidrug-resistant pneumococci in the post-vaccine era (2, 26, 27).

Hence, the benefit of the new PCV15 and PCV20 would be an increased potential to prevent pneumococcal cases by vaccine serotypes irrespective of their susceptibility pattern, but also to prevent strains with reduced susceptibility to antibiotics, thereby controlling antibiotic resistance (9, 28). Nevertheless, the prevalence and antibiotic resistance of serotype 24F—which is not covered by either of the two recently approved and commercially available PCVs—warrants particular attention. The increase in antimicrobial resistance to pneumococcus in recent years is a major public health problem. This increase was mainly due to the replacement of non-PCV serotypes as a cause of infection in children. Surveillance in the trends of these pneumococcal serotypes and the introduction of broader PCVs in children are the best strategies to detect the rise of resistant strains and prevent their spread.

The non-vaccine serotype with the greatest increase in IPD-RSP cases in the PCV13 sera was serotype 24F. This serotype poses a serious issue not only due to its association with antibiotic resistance and because it is not included in currently approved vaccines for children, but also because this serotype has a high meningeal tropism (29, 30). A new adult-focused vaccine candidate has been approved recently by EMA and FDA that includes 21 serotypes (PCV21) with high burden of disease in adults and has serotype 24F included (31). The implementation of PCV21 in adults may potentially contribute to the indirect control of serotype 24F in children through herd immunity.

During the first months of the COVID-19 pandemic in Spain, in the first half of 2020, the antibiotic exposure of pneumococcus by beta-lactams and macrolides increased. This was due to empirical antibiotic treatment of possible bacterial superinfection (32, 33). In this study, there is no clear impact of this antibiotic pressure. It should be considered that this exposure to beta-lactams and macrolides occurred in the adult population; therefore, no significant change in antibiotic resistance would be expected in the pediatric population.

### Conclusion

Since the introduction of PCV13, the overall incidence of IPD in the pediatric population declined substantially. However, this decrease has been less evident among isolates non-susceptible to penicillin or resistant to erythromycin. The PCV13 has not altered the MIC_90_ values of pediatric isolates causing IPD. A notable reduction in PCV13 serotypes, accompanied by an increase in non-PCV serotypes with reduced antimicrobial susceptibility, has been reported following vaccine implementation. Ongoing serotype surveillance remains essential to guide the formulation of next-generation PCVs and represents a key strategy to further reduce the burden of severe pneumococcal disease and mitigate the development of antimicrobial resistance in *S. pneumoniae*.

## MATERIALS AND METHODS

### Study design

This is a national longitudinal study including all pneumococcal strains in children under 15 years of age that were received at the Spanish Pneumococcal Reference Laboratory (SPRL) during the period 2009–2023. The SPRL receives pneumococcal isolates from Spanish hospitals through a passive surveillance system with minimal required information accompanying isolates (hospital and city, sample, age of the patient and date of isolation). According to the European Center for Disease Control (ECDC) data, the SPRL annually notifies of all IPD cases received using a passive surveillance system that covers 80% of the national level according to estimates by the National Center for Epidemiology reported to ECDC13. IPD-RSP and IPD-ER were analyzed separately. Finally, we included 4,455 total clinical isolates (1,458 IPD-RSP, 1,304 IPD-ER, and 1,693 isolates susceptible to both antibiotics) analyzing six different periods including 2009 (pre-PCV13 period), 2010–2012 (early PCV13 period), 2013–2016 (middle PCV13 period), 2017–2019 (late PCV13 period), 2020–2021 (COVID-19 period), and 2022–2023 (reopening period). Meningitis isolates were excluded from the study.

### Serotype characterization and antibiotic susceptibility

Serotyping was performed by Quellung reaction, dot blot assay using specific antisera from the Statens Serum Institut (Copenhagen, Denmark) and/or by PCR sequencing (2, 6).

For antimicrobial susceptibility, we tested penicillin, amoxicillin, cefotaxime, erythromycin, tetracycline, and levofloxacin. Antibiotic susceptibility was performed by disc-diffusion test and by microdilution broth test using Sensititre panels, and the MIC was interpreted using EUCAST breakpoints version 14. We considered IPD-RSP to be strains with MIC ≥ 0.12 µg/mL to penicillin and IPD-ER to strains with MIC ≥ 0.5 µg/mL to erythromycin (Table S3).

### Statistical analysis

We calculated the annual incidence of IPD as the number of IPD episodes per 100,000 population and year using population data from the Spanish National Statistical Institute as denominator. The corrected incidence was calculated by applying the population capture of 80% to the denominator. The MIC_90_ of all isolates, IPD-RSP strains, and IPD-ER strains was calculated in the different defined vaccination periods. We analyzed the changes in IPD cases after the COVID-19 pandemic. Comparison of different periods was analyzed by calculating the IRRs (cases, population, and incidences) using Poisson regression models. For each IRR, 95% confidence intervals were calculated. An IRR greater than 1 indicates a higher incidence rate in the comparator or exposed group, while an IRR less than 1 (but greater than 0) indicates a lower incidence rate in the comparator or exposed group. Chi-square test was used to compare the distribution of susceptible, susceptible with increased exposure, and resistant isolates across periods. Finally, the percentage of coverage of the new PCV15 and PCV20 vaccines was calculated for total IPD, IPD-RSP, and IPD-ER. Data were analyzed with GraphPad Prism 9, Microsoft Excel, and STATA v.14.

## SUPPLEMENTAL MATERIAL

Supplemental tables aac.01530-25-s0001.docx Tables S1 to S3.

ASM does not own the copyrights to Supplemental Material that may be linked to, or accessed through, an article. The authors have granted ASM a non-exclusive, world-wide license to publish the Supplemental Material files. Please contact the corresponding author directly for reuse.
