# Supplementary material for: Emergence of antibiotic-resistant pneumococcal serotypes causing invasive pneumococcal disease in children, Spain
Source: Antimicrob Agents Chemother. 2025 Dec 31;70(2):e01530-25. doi: 10.1128/aac.01530-25 (PMC12888898; doi:10.1128/aac.01530-25)
Supplement: Supplemental tables — Tables S1 to S3. [file aac.01530-25-s0001.docx]

**Emergence of antibiotic-resistant pneumococcal serotypes causing Invasive Pneumococcal Disease in children, Spain.**

Joaquín Llorente^1,2,3±^, Julio Sempere^1,4±^, Mirella Llamosí^1,4^, Covadonga Pérez-García^1^, Aída Úbeda^1^, Erick Joan Vidal-Alcántara^1^, Juan Carlos Sanz^5,6^, Mirian Domenech^1,4*^ and Jose Yuste^1,4^.

**Table S1: MIC₉₀ (µg/mL) of all isolates and strains exhibiting reduced susceptibility to penicillin and resistance to erythromycin from pediatric IPD cases across the different study intervals.**

**Table S2**: **Changes of the MIC_90_ (µg/mL) of the most prevalent serotypes in the different study periods.**

**ND, Not determined due to a sample size**

**Table S3: MIC (µg/mL) breakpoints for *S. pneumoniae* considered in the study following EUCAST criteria.**

| **MIC** | **S≤** | **SIE** | **R˃** |
| --- | --- | --- | --- |
| **Penicillin (indications other than meningitis)** | **0.06** | **0.06 - 2** | **2** |
| **Tetracycline** | **1** |  | **1** |
| **Chloramphenicol** | **8** |  | **8** |
| **Erythromycin** | **0.25** |  | **0.25** |
| **Cefotaxime (indications other than meningitis)** | **0.5** | **0.5-2** | **2** |
| **Amoxicicllin (indications other than meningitis)** | **0.5** | **0.5-1** | **1** |
| **Levofloxacin** | **0.001** | **0.001-2** | **2** |

**R: resistant, SIE: susceptible at increased exposure, S: susceptible.**
